# Ocular surface disease following LASIK and cataract surgery: a review of their interrelated complications

**DOI:** 10.3389/fmed.2025.1623324

**Published:** 2026-01-12

**Authors:** Matthew D. Spangler, Nila Kirupaharan, John D. Sheppard

**Affiliations:** 1 Drexel University College of Medicine, Philadelphia, PA, United States; 2 Virginia Eye Consultants, Norfolk, VA, United States; 3 Macon & Joan Brock Virginia Health Sciences at Old Dominion University, Norfolk, VA, United States

**Keywords:** artificial intelligence, cataract, dry eye disease, LASIK, surgery

## Abstract

**Background:**

Ocular surface disease is a multifactorial condition that is very commonly caused by dry eye disease (DED). Ophthalmic procedures intended to improve visual outcomes, laser-assisted *in situ* keratomileusis (LASIK) and cataract surgery, can paradoxically cause or exacerbate underlying ocular surface disease. This results in worsening vision and quality of life.

**Areas covered:**

This review examines the pathophysiological mechanisms contributing to ocular surface disease development following LASIK and cataract surgery. Both procedures are associated with the transection of corneal nerves, leading to decreased tear production, surface instability, altered neurotrophin production, and impairment of the blink reflex. Furthermore, these incisional procedures stress ocular tissues, while the use of preservatives in topical medications administered during or after these procedures may exacerbate dry eye symptoms. Management strategies for postoperative dry eye disease include, but are not limited to, the use of artificial tears, anti-inflammatory agents such as cyclosporine A or lifitegrast, nerve growth factor therapies, and procedural interventions including punctal plugs, thermal pulsation, lid exfoliation, and intense pulsed light therapy. Additionally, artificial intelligence has emerged as a promising tool to enhance the selection of optimal candidates, thereby minimizing risk and optimizing postoperative outcomes and improving patient satisfaction.

**Discussion:**

LASIK and cataract surgery are highly effective vision correction procedures that have constantly evolved to improve visual outcomes and mitigate side effects. However, ocular surface diseases remain a common complication affecting the outcome and quality of life of post-operative patients. The integration of personalized treatment and AI-based screening protocols can help improve patient outcomes.

## 1 Introduction

Ocular surface disease is a multifactorial condition characterized by symptomatic discomfort, visual disturbances, and tear film instability, often driven by environment, inflammation, meibomian gland dysfunction, and chronic exposure to topical preservatives (1). The most prevalent cause of ocular surface disease globally is dry eye disease (DED), as it affects an estimated 5 million individuals aged 50 years and older in the United States alone (2, 3). Notably, it has a disproportionately higher prevalence of women, particularly those with menopausal hormonal changes (2). Schaumberg et al. (2) reported that up to 7% of all U. S. women experience DED symptoms that are clinically relevant; the prevalence of this increases with age and systemic comorbidities. Beyond its commonality, DED poses a burden to functional vision and quality of life, impacting everyday activities like reading, driving, computer use, and any visual tasks (3). Some of the symptoms these patients face are chronic discomfort, fluctuating vision, and photophobia, contributing to a significant impact on patients every day life and psychosocial status (3). Therefore, we must examine what is causing this and how to address it.

The pathophysiology of DED is multifactorial and is characterized by a combination of disruption in tear film homeostasis, tear film instability, hyperosmolarity, ocular surface inflammation, and tissue damage (3–7). The two most common subtypes of DED include aqueous-deficient DED, accounting for approximately 10–25% of cases, and evaporative DED, which comprises over 85% of cases (4–6, 8). The most common cause of evaporative DED is meibomian gland dysfunction (MGD), which results in a decrease in lipid secretion from these glands, promoting the tear film instability (5, 8). Without a proper lipid coating on the eye, the aqueous component evaporates more quickly, creating a constant cycle of dry eye. Other factors that contribute to DED include aging, environmental exposures, systemic autoimmune diseases, contact lens wear, digital device use, pharmacological agents, hormonal influence, and neuropathic ocular pain (6, 7). Immune dysregulation involves T-cell activation, cytokine release, and chronic epithelial stress that results in the initiation and perpetuation of this disease process (4). In recent years, increasing emphasis has been placed on the need for standardized diagnostic criteria and personalized treatment strategies, owing to the heterogeneous presentation of DED and its substantial impact on patient quality of life. Notably, DED may arise as a complication of two common ophthalmic procedures, laser-assisted *in situ* keratomileusis (LASIK) and cataract surgery.

LASIK is an elective refractive procedure aimed at correcting visual impairments such as myopia, hyperopia, and astigmatism. Although 38 to 75% of patients undergoing LASIK present with pre-existing dry eye symptoms, postoperative DED has been reported in up to 95% of cases (9–13). The pathogenesis of LASIK-associated DED is primarily attributed to the creation of a corneal flap, which transects corneal nerves, resulting in decreased corneal sensation, altered neurotrophin production, and impaired lacrimal reflexes. This neural disruption contributes to reduced tear production and exacerbation of ocular surface disease (14). Management strategies for post-LASIK DED range from first-line therapies such as artificial tears to more advanced interventions, including topical immunomodulatory agents such as cyclosporine A 0.05% or lifitegrast 5% (14, 15).

Similarly, cataract surgery, a highly effective and increasingly common procedure due to the aging global population, has been associated with the onset or exacerbation of DED (16, 17). While procedural advancements have primarily focused on mitigating sight-threatening complications, less attention has been paid to postoperative ocular surface disorders, which can significantly impair patient-reported outcomes and quality of life (16). Post-cataract surgery DED occurs in 10 to 33% of patients (17–19). Some contributing factors include prolonged use of postoperative antibiotic and corticosteroid eye drops, incision-related ocular surface irregularities leading to reduced tear film stability, diminished corneal sensitivity resulting in decreased tear secretion, and procedure-induced inflammation impairing tear production and stability (20). Management of DED in this context aligns with that for post-LASIK cases, encompassing both conservative modalities and more advanced therapeutic options, including topical anti-inflammatory agents and procedural interventions (17).

Logically, the same principles guiding detection and treatment of OSD and DED in LASIK patients most certainly apply to other corneal procedures, including PRK (photorefractive keratectomy), PTK (phototherapeutic keratectomy), LASEK (laser epithelial keratomileusis), SMILE (small incision lenticule extraction), lamellar keratectomy and the wide range of corneal transplantation procedures now commonly performed. Additionally, a broad range of lens-based procedures endure similar ocular surface stresses compared to standard cataract surgery. These include IOL (intraocular lens) exchange, refractive lens exchange (RLE), IOL repositioning, sutured IOL surgery, and ICL (intraocular collamer lens) implantation.

Herein, this paper will examine the most performed surgery worldwide, cataract surgery, and the most common vision correction surgery, LASIK, to examine how these common everyday surgeries impact patients’ ocular surface (10, 16). Despite the significant visual improvements these surgeries can have, one must acknowledge the side effects that impact the visual outcome. The potential for DED to develop or worsen postoperatively represents a significant paradox. Accordingly, this review aims to elucidate the mechanisms underlying the development of DED following LASIK and cataract surgery and explore evidence-based management and prevention approaches.

## 2 Ocular surface disease following ocular surgery

### 2.1 Pathophysiology of LASIK-induced complications

Among ophthalmic procedures, laser-assisted *in situ* keratomileusis (LASIK) surgery is strongly associated with the development of DED (21). Wilson et al. (22) were the first to describe the mechanism of LASIK-induced neurotrophic epitheliopathy, observing that patients developed corneal epithelial erosions and denervation of corneal nerves, impairing tear production, tear secretion, and the blink reflex. The procedure involves three main steps: corneal flap creation using either a microkeratome or a femtosecond laser, followed by excimer laser stromal ablation to correct the visual acuity, and repositioning of the flap; each of these steps affects corneal homeostasis.

The primary pathophysiological mechanism underlying LASIK-induced dry eye disease (DED) is the reduction in corneal sensitivity resulting from the creation of the corneal flap. Corneal nerve plexus enters the cornea from the periphery in a radial fashion, forming the subepithelial and sub-basal nerve plexuses located beneath Bowman’s membrane. Most notably, the long ciliary nerves enter the cornea at the 3 and 9 o’clock positions, impairing corneal sensitivity, the blink reflex, and feedback to the lacrimal functional unit (21–24). The LASIK procedure transects this tissue layer circumferentially, sparing only the hinge (24). Deciding upon hinge placement is important as superior hinges typically sever more corneal nerves than nasal hinges; therefore, superior hinge placement correlates with greater reduction of corneal sensation and higher post-operative rates of DED (25, 26).

The neurotrophic state, termed LASIK-induced neurotrophic epitheliopathy, compromises epithelial barrier function, increasing the susceptibility to tear film instability, resulting in delayed healing (22, 27). This instability is perpetuated by decreased blink rate from non-functioning neuro-feedback loops, post-operative inflammation, and surgical manipulation, which further impairs meibomian gland function, reducing lipid secretion and destabilizing the tear film, which in turn increases tear evaporation (21, 27–29). In cases where symptoms of dry eye persist chronically, incomplete or aberrant reinnervation of the corneal nerves is believed to be a significant contributing factor.

LASIK-associated dry eye typically resolves within 6 months postoperatively, coinciding with the reinnervation of the LASIK flap (22). However, in a minority of patients, chronic dry eye may persist for longer than one year. Several patient- and procedure-related risk factors have been associated with an increased likelihood of developing persistent DED following LASIK. These include pre-existing dry eye disease, female sex, Asian ethnicity, higher degrees of refractive correction, greater ablation depth, thicker corneal flaps, and flap configurations (13, 25, 26, 30–32). Yahalomi et al. (33) showed that PRK and LASEK dry eye symptoms were less severe 6 months post-operatively than LASIK. Higher dry eye risk was associated with female sex and higher corrections, but not patient age (33). Fortunately, this outcome is relatively rare, with an incidence of approximately 0.8% (23).

Despite ongoing efforts within the ophthalmic community to standardize the diagnosis of DED, considerable variability remains in clinical practice. Common objective diagnostic modalities include tear break-up time (TBUT), Schirmer’s test, tear film osmolarity assessment, and ocular surface staining techniques (34). Overall, LASIK alters multiple levels of ocular surface physiology, corneal innervation, epithelial integrity, tear film composition, and blink mechanics, creating a multifactorial pathway through which postoperative DED can arise.

### 2.2 Alternative to LASIK

A novel procedure for refractive correction, small incision lenticule extraction (SMILE), was developed to mitigate complications associated with traditional LASIK surgery, one of the most notable being the high prevalence of postoperative dry eye disease (DED). SMILE is a flapless technique in which a femtosecond laser creates an intrastromal lenticule through a small vertical incision, thereby minimizing disruption to the corneal nerve plexus (14). The incision size in SMILE is typically 3–4 mm chord length, significantly smaller than the 8–9 mm diameter, ~25 mm circumference flap created in LASIK procedures (24).

Liu et al. and Shen et al. (35, 36) demonstrated that there was no statistically significant difference in long-term visual acuity outcomes between SMILE and LASIK, suggesting that the newer procedure maintains comparable efficacy in vision correction. However, Shen et al. (36) reported that patients undergoing LASIK experienced significantly more dry eye symptoms, as evidenced by higher Ocular Surface Disease Index (OSDI) scores and decreased corneal sensitivity when compared to those who underwent SMILE. Similarly, Wang et al. (37) found that SMILE was associated with significantly better tear break-up time (TBUT), indicating a more stable tear film and potentially reduced dry eye symptoms postoperatively. Nevertheless, DED remains a potential complication following SMILE, as some degree of corneal nerve plexus disruption and inflammatory marker upregulation, such as increased levels of interleukin-6 (IL-6), can still occur (24).

### 2.3 Pathophysiology of cataract surgery-induced complications

Cataract surgery is among the most commonly performed surgical procedures in modern medicine, boasting a remarkable safety profile. However, like all surgical interventions, it is not without potential complications. A variety of techniques are employed in cataract surgery, including standard phacoemulsification, small incision cataract surgery (SICS), extracapsular cataract extraction (ECCE), and femtosecond laser-assisted cataract surgery (FLACS), among others (18). Postoperative DED is a well-recognized complication. Increased surgical duration, microscopic light exposure, and prolonged phacoemulsification time are associated with higher DED risk (17, 38). Notably, FLACS has been shown to further elevate the risk of DED when compared to other techniques (17, 39–42).

The major steps of the cataract surgery procedure include creation of a corneal incision, capsulorhexis, ultrasonic emulsification of the lens, aspiration of lens fragments, and intraocular lens implantation. Similar to laser-assisted *in situ* keratomileusis (LASIK), the pathogenesis of ocular surface disease following cataract surgery involves incision-related disruption of the tear film, diminished corneal sensitivity, disruption of the lacrimal functional unit, reduced reflex tear secretion, and reduced blink efficiency (17, 38, 40, 42). The creation of this tear film instability can also be secondary to astigmatic limbal relaxing incisions commonly performed during cataract surgery (38). However, DED post-operatively for cataract surgery also places a focus on exacerbated inflammation induced by surgical manipulation, ocular surface desiccation, and exposure to topical medication with preservative; these processes all lead to increased goblet cell loss, epithelial apoptosis, and tear film instability (43–47).

Surgical manipulation and ocular surface desiccation disrupt the balance of the tear film by directly affecting its three principal layers, lipid, aqueous, and mucin, and the ocular surface cells that maintain them. Mechanical trauma to the corneal and conjunctival epithelium during incisions, phacoemulsification, or laser ablation can damage superficial epithelial cells and reduce the density and function of conjunctival goblet cells, which secrete mucins critical for tear film stability (38, 43, 44). This compromise of the mucin layer leads to poor tear adhesion to the corneal surface by increasing the aqueous tears’ surface tension, resulting in accelerated tear break-up and increased exposure of the epithelium to environmental stress. In addition, exposure to air, bright surgical microscope light, and irrigation fluids during surgery can desiccate the ocular surface, thinning the aqueous layer and altering lipid layer distribution, further increasing evaporation and destabilizing the tear film (38, 40).

The use of preserved topical medications, both intraoperatively and postoperatively, can exacerbate ocular surface inflammation. Commonly used agents such as sodium hyaluronate 0.1%, diquafosol, and nonsteroidal anti-inflammatory drugs (NSAIDs) have somewhat surprisingly been implicated as DED contributors. Proposed mechanisms include epithelial apoptosis, ocular surface damage, and decreased goblet cell density (17, 43–45, 48). In contrast, preservative-free formulations may offer protective effects due to superior antioxidant properties, which help reduce postoperative inflammation (46). Topical NSAIDs may contribute to DED by inhibiting prostaglandin E2 (PGE2), a molecule that supports mucous epithelial metaplasia and proliferation (47). Furthermore, inadequate irrigation during surgery can result in direct ocular surface desiccation trauma, thereby increasing the likelihood of DED (16). Additionally, the prolonged postoperative use of antibiotic-steroid eye drops plays a significant role in the disease process (20). Lastly, immunomodulatory agents such as interferon-gamma (IFN-*γ*), used perioperatively in some contexts, may promote goblet cell loss and elevate pro-inflammatory cytokines in the tear film, thereby contributing to lacrimal gland dysfunction and ocular surface inflammation (49).

Interestingly, femtosecond laser-assisted cataract surgery, while providing precise capulotomy and lens fragmentation, may increase the risk of postoperative DED due to occasionally longer operative times, greater energy delivery, and increased ocular surface exposure compared to convention phacoemulsification (40–42). Overall, cataract surgery affects multiple aspects of ocular surface physiology, creating a multifactorial pathway for the development of post-operative DED. This highlights the importance of post-operative follow-up with patients, proper assessment for DED with or without symptoms present, and perioperative ocular surface optimization.

## 3 Treatments for ocular surface disease

Effective management of dry eye disease (DED) preceding and following LASIK is critical. Albeitz et al. (50) identified a significant association between chronic post-LASIK DED and refractive regression, underscoring the importance of early and appropriate intervention. Attempts to improve visual acuity through enhancement surgeries in patients with untreated DED may further exacerbate ocular surface instability and worsen visual disturbances (22).

First-line treatment typically involves the use of artificial tears and ocular emollients, which aim to lubricate and stabilize the tear film, thereby addressing symptomatic discomfort (14). Beyond symptomatic relief, more targeted pharmacologic therapies have been developed to address the underlying inflammatory and nerve degeneration pathways of the disease. Diquafosol, for instance, is a P2Y2 receptor agonist that increases intracellular calcium ion concentrations, thereby stimulating mucin and lipid secretion to enhance tear film stability (14). Additionally, diquafosol has been shown to promote corneal epithelial healing by accelerating cell migration and proliferation via activation of the epidermal growth factor receptor/extracellular signal-regulated kinase (EGFR/ERK) pathway (51).

Cyclosporine A 0.05% has demonstrated superior efficacy in treating post-LASIK DED compared to diquafosol and artificial tears, significantly improving tear break-up time (TBUT) and Ocular Surface Disease Index (OSDI) scores (14, 52–55). Cyclosporine exerts its effects through multiple mechanisms, including increasing goblet cell density, enhancing aqueous tear production, and promoting corneal nerve regeneration, thereby restoring corneal sensitivity (14, 56). In addition, nerve growth factor (NGF) has emerged as a promising therapeutic agent by facilitating faster reinnervation of the corneal epithelium and improving sensory function (57, 58). These treatments allow for a more personalized approach to the treatment of LASIK-induced dry eye disease by focusing on treating the underlying mechanism of corneal denervation.

The treatment of ocular surface disease following cataract surgery parallels the management strategies used for post-LASIK dry eye, beginning with conservative therapies and progressing to pharmacological treatments aimed at modulating the inflammatory response. Cyclosporine 0.1% has demonstrated efficacy, even when used prophylactically, in reducing inflammatory markers implicated in the development of postoperative dry eye disease (DED) (59). Key strategies to mitigate the risk of DED include the use of preservative-free medications, minimizing the use of topical nonsteroidal anti-inflammatory drugs (NSAIDs) and interferon-gamma, and ensuring appropriate irrigation techniques during surgery (16, 17, 43–49). Hovanasian et al. (60) conducted a study that found patients who used Lifitegrast for 28 days before they had preoperative biometry conducted for cataract surgery had a significantly greater accuracy in preoperative biometry and postoperative outcomes than those who did not use Lifitegrast before biometry. Furthermore, Fogagnolo et al. (61) demonstrated that supplementation of standard hyaluronic acid-based lubricating eye drops with *Ginkgo biloba*, a known antioxidant, significantly improved dry eye symptoms in the postoperative setting. Overall, a preventative approach that emphasizes reducing ocular surface irritation is central to minimizing postoperative dry eye complications following cataract surgery.

Other adjunctive treatments for post-ocular surgery DED include punctal plugs to reduce tear drainage, dietary supplementation with omega-3 fatty acids, eyelid-warming devices to improve meibomian gland function, and advanced cash based procedural technologies such as thermal pulsation and intense pulsed light therapy (14).

## 4 Discussion

Pre-existing ocular surface disease (OSD), particularly dry eye disease (DED), presents significant challenges in the preoperative evaluation and postoperative outcomes of ocular surgery (62). Table 1 can be used as a reference to compare factors between LASIK and cataract surgery with respect to OSD complications. OSD can cause tear film instability and irregular corneal surfaces, leading to inaccurate keratometry and biometry measurements so crucial to accurate intraocular lens (IOL) power calculations (63, 64). Corneal staining, punctate epithelial erosions, and irregular astigmatism may further compromise refractive predictability and visual outcomes (65). Additionally, epithelial and conjunctival changes associated with OSD can mask subtle underlying pathology, such as early limbal stem cell deficiency or conjunctivochalasis, which may influence surgical risk and outcomes if not recognized preoperatively (62, 66). Surface abnormalities may also limit candidacy for elective procedures such as LASIK or premium IOL implantation, withholding intervention from deserving patients. Optimization of the ocular surface prior to surgery using anti-inflammatory therapy, tear substitutes, and treatment of underlying meibomian gland dysfunction has been shown to improve measurement accuracy and reduce postoperative complications (62, 64). Without adequate preoperative management, patients are at increased risk of delayed epithelial healing, postoperative inflammation, and ocular discomfort, all of which can lead to reduced satisfaction and suboptimal visual results (62, 66). Given this bidirectional influence, proactive identification and treatment of OSD prior to ocular surgery is essential to optimize outcomes.

**Table 1:** Comparison of mechanisms, risk factors, and outcomes related to dry eye disease in LASIK and cataract surgery procedures.

Category	LASIK	Cataract surgery
Primary mechanism of postoperative DED	Creation of corneal flap transects sub-basal nerve plexus → reduced corneal sensation and impaired lacrimal feedback loop (21, 22, 24) Decreased blink rate and meibomian gland dysfunction contribute to evaporative DED (27, 28)	Corneal incisions, including limbal relaxing incisions (LRIs), disrupt corneal nerves and ocular surface integrity (17, 18) Microscope light exposure, inflammation, and ocular surface desiccation impair tear film stability (16, 38) Postoperative topical medications and preservatives contribute to goblet cell loss and inflammation (20, 43–45, 48)
Additional contributing factors	Ablation depth increases nerve damage (25, 26, 31, 32) Pre-existing tear film instability	LRIs and longer surgical duration increase risk (17, 38) Poor irrigation or prolonged phaco time increases epithelial injury (16) Pre-existing tear film instability
Common postoperative pathophysiology	Neurotrophic epitheliopathy due to denervation (22) Reduced tear secretion and increased osmolarity	Reduced goblet cell density and mucin production (43–48) Tear film instability due to epithelial injury and inflammation
Incidence of postoperative DED	Short-term DED in up to 95% of patients (9–13) Chronic DED beyond 1 year occurs in ~0.8% (23)	DED occurs in 10–33% of cases (17–19) New-onset DED reported in up to 37.4% even in asymptomatic patients (48)
Key patient-related risk factors	Pre-existing DED, female sex, Asian ethnicity (13, 30) High refractive correction	Older age (primary cataract population). Pre-existing DED or MGD Autoimmune disease or systemic medication use
Procedure-related risk factors	Thick flaps Superior hinge position Greater stromal ablation	Long phacoemulsification time FLACS increases risk of postoperative DED (17, 39–42) Preserved medications used perioperatively (43, 44, 46–48)
Clinical course	Typically resolves within 6 months as nerve fibers regenerate (22)	May persist for several months; severity depends on surgical factors and topical regimen
Typical symptoms	Burning, foreign body sensation, fluctuating vision, photophobia	Ocular irritation, fluctuating vision, foreign body sensation; may affect visual rehabilitation
Diagnostic features	Reduced corneal sensitivity, lower TBUT, elevated OSDI, increased osmolarity	Increased corneal staining, reduced Schirmer scores, unstable tear film
Management strategies	Artificial tears (preservative free), diquafosol, cyclosporine 0.05%, nerve growth factor therapy (51–58) MGD treatment: warm compress, IPL, thermal pulsation	Preservative-free artificial tears preferred. Cyclosporine 0.1% prophylactically reduces inflammation (59) Avoid unnecessary NSAIDs; consider antioxidants like *Ginkgo biloba* (61)
Long-term outcomes	Excellent visual outcomes when DED is treated early; untreated DED may contribute to refractive regression (50)	DED can delay rehabilitation and reduce patient satisfaction with IOL visual outcomes (62–66)

Ocular surgery is a well-recognized contributor to ocular surface disease (OSD), with cataract surgery and refractive procedures particularly implicated in both the development and worsening of DED (66). Surgical incisions can damage corneal nerves, disrupt the lacrimal functional unit and reduce reflex tear secretion, while intraoperative trauma—such as prolonged exposure to bright microscope light, mechanical irritation, and use of antiseptics or anesthetics—can damage the corneal and conjunctival epithelium, decrease goblet cell density, and destabilize the tear film (48, 67, 68). Postoperative inflammation, especially in the presence of preserved topical medications, may prolong epithelial healing and exacerbate surface damage (17, 69). Clinical studies have shown that new-onset DED occurs in up to 37.4% of patients following cataract surgery, even in those without prior symptoms. This DED can persist for several months postoperatively, with tear film instability, reduced Schirmer scores, and increased corneal staining commonly observed (17, 48). Additionally, patients with preexisting OSD are at greater risk for prolonged recovery and reduced satisfaction after surgery (62, 66). While cataract surgery is most frequently associated with postoperative DED, other anterior segment procedures—including LASIK, trabeculectomy, and vitrectomy—can similarly compromise the ocular surface, particularly those involving full-thickness corneal incisions (66). Older adults, who comprise the surgical demographic, are especially vulnerable due to slowly deteriorating baseline tear dysfunction (70). Thus, thorough preoperative assessment and proactive management of the ocular surface, including lubrication, anti-inflammatory therapy, and treatment of meibomian gland dysfunction, are essential to mitigate postoperative complications and support visual rehabilitation (48, 67, 71).

Managing complications to LASIK and cataract surgery in the future will most likely evolve to incorporate technological advances, for example, artificial intelligence (AI). AI is rapidly transforming ophthalmology, with notable advancements not only in the diagnosis and management of DED, but with the emerging applications in refractive and cataract surgery. AI-driven systems have demonstrated high accuracy in evaluating the tear film through classification of DED subtypes, assessing disease severity, and analyzing diagnostic metrics such as meibography, tear meniscus height, and corneal staining patterns (72–74). Although integration of AI into refractive surgery remains early, machine learning tools are beginning to streamline preoperative screening by identifying contraindications and have shown the potential to outperform ophthalmologists in predicting outcomes; this ultimately could improve patient selection, counseling, and postoperative results of patients (75–79). Similarly, AI is increasingly used in cataract care, contributing to automated diagnosis, IOL power calculations, and biometric assessments; however, its application to post-cataract ocular surface complications is still limited (80–84). As AI models become more developed, the incorporation of surgical parameters, for example, incision characteristics, operative duration, and exposure to toxic agents to the epithelium, in addition to patient-specific ocular surface metrics, could enable the precise prediction of postoperative DED risk. Overall, the continued integration of AI across refractive and cataract surgery pathways offers a future in which individualized surgical planning, enhanced screening accuracy, and proactive identification of high-risk patients may significantly reduce postoperative ocular surface morbidity and improve long-term visual outcomes.

In conclusion, ocular surface disease remains a prevalent and clinically significant complication following elective LASIK and cataract surgery. A comprehensive understanding of the distinct pathophysiologic mechanisms underlying postoperative DED is essential for the development of effective preventive and therapeutic strategies. Advances in pharmacologic treatments, procedural interventions, and emerging pharmacologic and artificial intelligence technologies hold considerable promise in improving early identification of at-risk patients and optimizing individualized postoperative management. Ongoing research efforts will be crucial to further mitigate the incidence and severity of ocular surface disease, ultimately enhancing visual outcomes and patient quality of life following LASIK and cataract procedures.
